# Defining short and prolonged breath-holds

**DOI:** 10.1259/bjr.20200191

**Published:** 2020-06-02

**Authors:** Michael Parkes, Jason Cashmore, Stuart Green, Thomas Clutton-Brock, Irma Van Dijk, Zdenko van Kesteren, Geertjan van Tienhoven, Arjan Bel, Joost van den Aardweg, Markus Stevens

**Affiliations:** 1 School of Sport, Exercise and Rehabilitation Sciences & NIHR/Wellcome Trust Clinical Research Facility, Queen Elizabeth Hospital Birmingham, University of Birmingham, Birmingham, United Kingdom; 2 Department of Medical Physics, University Hospitals Birmingham NHS Foundation Trust, Birmingham, West Midlands, United Kingdom; 3 Department of Critical Care Medicine, University Hospitals Birmingham NHS Foundation Trust, Birmingham, United Kingdom; 4 Department of Radiation Oncology, Amsterdam University Medical Center, location AMC, University Hospitals Birmingham NHS Foundation Trust, Birmingham, United Kingdom; 5 Department of Respiratory Medicine, Amsterdam University Medical Center, location AMC, Meibergdreef 9, Amsterdam, the Netherlands; 6 Department of Anesthesiology, Amsterdam University Medical Center, location AMC, Meibergdreef, Amsterdam, the Netherlands

In Khot et al.’s interesting recent paper in the *British Journal of Radiology*,^1^ they describe breath-hold durations of 36 s with maximum inspirations of air as “prolonged breath-holding”. Breath-holding is indeed increasingly applied to mitigate the effects of respiratory motion, both in radiology to improve the imaging and in radiotherapy to decrease the margins. In breast cancer radiotherapy,^2^ it is also used to expand the lung volume to avoid irradiation of the heart. Here, the breath-holds with air are described as multiple (5–10), “short” breath-holds (20–30 s) and are sometimes called deep inspiratory breath-holds.

For radiotherapy, we have demonstrated the feasibility of 60 s breath-holds of air to immobilize the pancreas.^3^ Furthermore, we have also developed techniques involving preoxygenation and hypocapnia to enable breast cancer patients to achieve safely “prolonged” breath-holds (>5 min),^4^ and for healthy volunteers to perform multiple “prolonged” breath-holds (nine successive breath-holds of >4 min).^5^

The descriptions of “short” and “prolonged” breath-holds are becoming increasingly confusing. It seems therefore important to adopt consistent terminology for medical imaging and radiotherapy.

We propose that breath-holds achieved with air (durations typically less than 1 min and best measured in the order of seconds) are described as “short”.^1–3^ Whereas breath-holds achieved with preoxygenation and hypocapnia (durations best measured in the order of minutes) are described as “prolonged”.^4,5^

## Authors' reply re: defining short and prolonged breath-holds

^1^Rachita Khot MD, ^2^Melissa McGettigan MD, ^3^James Patrie MS and Sebastian Feuerlein MD

^1^Department of Radiology & Medical Imaging, University of Virginia Health System 1215 Lee Street Charlottesville, Charlottesville, VA 22908

^2^Department of Radiology, H. Lee Moffitt Cancer Center 12902 Magnolia Drive, Tampa, FL 33612

^3^Department of Public Health Sciences, University of Virginia Health System 1215 Lee Street, Charlottesville, VA 22908

### Reply to letter to the editor

We would like to thank Dr. Parkes and colleagues for their thoughtful remarks. The use of terms like “short” and “prolonged” breath-holding is certainly context-dependent. In the context of clinical imaging and for an average patient with possible comorbidities such as obesity, lung disease or heart failure a 30 s breath-hold is probably near the achievable maximum, especially if repeated breath-holds are required during the dynamic phase of a liver MRI. Most clinical MRI protocols therefore limit breath-holding to 15–20 s. In the context of radiation therapy, Dr. Parkes et al have done some fascinating research with preoxygenation and hypocapnia to achieve ultra-long breath-holds up to several minutes, so 30 s can indeed be considered “short”.

We believe that the context and focus of our paper, while experimental in design, is closer to clinical everyday imaging than experimental radiation therapy and while acknowledging the validity of the author’s general argument, we consider the terms used to describe the length of our breath holds appropriate.
